# Annotations

**Published:** 1890-01-11

**Authors:** 


### Annotations.

**Antiseptics v. Plain Water.**

Dr. C. R. Illingworth, whose experiments with biniodide of mercury are of a very valuable character, writes to controvert the somewhat prevalent idea that plain water is as good a dressing for ordinary wounds as a substance which is known to be antiseptic. Dr. Illingworth is emphatically right, as the highly scientific researches of Sir Joseph Lister have again and again convincingly proved. It is extremely desirable in the interests of surgery as an art, and still more desirable in the interests of the patients on whom the art is practised, that mischievous errors should be constantly exposed, and, as far as possible, banished, at any rate, from the region of daily work. One simple consideration ought to be sufficient to convince even the most sceptical surgeons of the risk and danger of using plain water for the dressing of open wounds. That consideration is, of course, this: that water is so very frequently impure and often even dangerously poisonous. If impure water taken into the alimentary canal can set up such diseases as diphtheria, typhoid fever, and the like, by mere absorption through the ordinary absorbents, and when there is no suspicion of a wounded or abraded surface, how much more certainly will impure water do incalculable mischief when poured upon or applied to a deep cut or other open wound! The character of any particular antiseptic is comparatively unimportant, provided it fulfils the two conditions of antisepsis and non-irritability. Of course, antiseptics are more or less potent according to their constitution, and are to be selected for particular cases from that standpoint. Dr. Illingworth pins his faith to the biniodide of mercury, and in it he considers he has found a surgical and medical philosopher's stone, which transmutes every diseased condition to which it is applied into the pure gold of perfect health. Whilst we are not prepared by personal experience to go so far as that in the biniodide faith, we are quite convinced of the soundness of his general contention, that in a suitable solution it is infinitely to be preferred to plain water as a dressing for open wounds. Dr. Illingworth dissolves the biniodide in iodide of sodium, and his ordinary solution has a strength of one in a thousand. He uses it in all kinds of wounds, abscesses, and sores, and finds that it prevents suppuration and ensures rapid union, in most cases of the "primary" kind. Moreover, he says that irritation of the skin with this dressing is a thing practically unknown. In characteristic phraseology, Dr. Illingworth adds that though wounds dressed in this way remain sweet, heal rapidly, and never suppurate, the same wounds dressed with plain water dressings "would stink in two days and suppurate in four." These facts deserve the careful consideration of all surgeons who still dress wounds with plain water.

**Missing Contributions to Charities.**

Mrs. Hilton, of the Crêche and Orphan Home in Stepney-causeway, writes to the daily papers to complain that several letters recently addressed to her, and containing contributions, have been lost in the course of transmission through the Post Office. One letter, containing a cheque, was lost. The donor waited a month, and then sent the amount in postal orders, which were also lost. It is feared that many contributions have been sent by benevolent persons who have not complained of not having received acknowledgments. There is a great probability that what has happened to Mrs. Hilton's Crêche is constantly happening to hospitals and other charities. It is not easy to see what hospital officials can do in the matter; but it is certain that in the interests both of the charities and of the public this apparently common and nefarious method of thieving should be promptly suppressed. Donors to the various charities are the proper persons to take the matter in hand. Not only should they observe the common precaution of "crossing" their cheques, but they should in all instances ask for acknowledgments by return of post. If such acknowledgments are not received they should communicate at once with the secretary, using envelopes of a different shape, addressing them in different handwriting, and posting them if possible in different districts or towns. Immediate and energetic measures may then be taken for the discovery of the thief or thieves. If all donors to charities will make this a matter of personal duty they will render an important public service, and probably suppress this kind of thieving for a long time to come.

**Directions for Influenza.**

The first thing to be done by any person who feels that he is probably going to be attacked by influenza, is to go to bed and send for the doctor. This is much wiser than attempting self-treatment; but there are certain things which the patient and his friends ought to do on their own behalf. Common-sense, for example, suggests that if a patient is sneezing, running at the eyes, and aching in every limb, it will be wise to warm the sheets of the bed a little before he is put into them. For this purpose, two or three hot bottles may be used; or, in the case of the poor who have not got hot bottles, hot bricks wrapped in flannel will serve the purpose. In any event, the patient should not be put into a cold, chill bed. Quite as important as the warming of the bed is the warming of the bedroom. It is astonishing, at any rate to a medical man, how little knowledge and homely sense people seem to have on such questions as the coldness or warmth of the air we breathe. They would never think of stripping a patient naked and making him stand with his skin exposed to the cold air of a cold bedroom for two or three hours unless they wanted to kill him; but they will put him into a fireless room with a Greenland atmosphere for days and weeks together, and then be astonished that he sneezes all day, coughs all night, and never gets a whit better in spite of the doctor and his physic. Let a nice fire then be lighted in the bedroom, and let the temperature, at a distance of ten or twelve feet from the fire, be maintained at about 65deg. F. Having now got the patient put to bed, the room well warmed, and the doctor sent for, what is next to be done? The doctor's orders are to be carried out to the letter. But what of those who cannot afford to call in the doctor? They, too, should go to a well-warmed bed in a well-warmed room. They should take hot, sustaining drinks—such as beef-tea, gruel, bread and milk, and so on—every two or three hours. If difficulty of breathing be experienced, linseed and mustard poultices may be applied to the chest and between the shoulders. Let everybody bear in mind, however, that if poultices are to be used they must be put on hot, kept hot, and changed frequently. When they are finally taken off, the chest and back must be rubbed dry with a dry hot towel. Then the parts from which the poultices have been removed must be covered with three or four folds of hot dry flannel. A mild aperient pill or a dose of compound liquorice powder may be taken at the commencement of the symptoms. If the depression be great small doses of the ammoniated tincture of quinine should be given every two or three hours. As soon as the appetite begins to return regular meals of light solid food are to be commenced. It is desirable to keep to the bedroom for two or three days. When convalescence is well established a regularly used sitting-room may be exchanged for the bedroom; but not a sitting-room that only has a fire in it now and then. After two or three days of the sitting-room the first dry day may be taken advantage of for one or two short airings outside. If care be exercised in sitting and sleeping in none but warmed rooms, and in avoiding chills outside, there will be no relapse. It is thus seen that with common-sense at the helm even the poor will have little difficulty in steering an influenza patient's barque safely through the storm.

